# Patterns of white matter hyperintensities associated with cognition in middle-aged cognitively healthy individuals

**DOI:** 10.1007/s11682-019-00151-2

**Published:** 2019-07-05

**Authors:** Anna Brugulat-Serrat, Gemma Salvadó, Carole H. Sudre, Oriol Grau-Rivera, Marc Suárez-Calvet, Carles Falcon, Gonzalo Sánchez-Benavides, Nina Gramunt, Karine Fauria, M. Jorge Cardoso, Frederik Barkhof, José Luis Molinuevo, Juan Domingo Gispert, for the ALFA Study, Jordi Camí, Raffaele Cacciaglia, Grégory Operto, Stavros Skouras, Carolina Minguillón, Albina Polo, Cristina Mustata, Laia Tenas, Paula Marne, Xavi Gotsens, Tania Menchón, Anna Soteras, Laura Hernandez, Ruth Dominguez, Sandra Pradas, Gema Huesa, Marc Vilanova, Sabrina Segundo, Jordi Huguet

**Affiliations:** 1 Barcelonaβeta Brain Research Center, Pasqual Maragall Foundation, Wellington 30, 08003 Barcelona, Spain; 2 School of Biomedical Engineering and Imaging Sciences, King’s College London, London, UK; 3 Dementia Research Centre, UCL, London, UK; 4 Centro de Investigación Biomédica en Red de Bioingeniería, Biomateriales y Nanomedicina (CIBER-BBN), Madrid, Spain; 5 Translational Imaging Group, CMIC, UCL, London, UK; 6 Brain Repair and Rehabilitation, UCL Institute of Neurology, London, UK; 7 Radiology & Nuclear Medicine, VU University Medical Centre, Amsterdam, the Netherlands; 8 Centro de Investigación Biomédica en Red de Fragilidad y Envejecimiento Saludable (CIBERFES), Madrid, Spain; 9 Universitat Pompeu Fabra, Barcelona, Spain

**Keywords:** White matter lesions, Magnetic resonance imaging, Preclinical Alzheimer’s disease, Cardiovascular risk, Hypertension

## Abstract

White matter hyperintensities (WMH) are commonly detected in the brain of elderly individuals and have been associated with a negative impact on multiple cognitive domains. We aim to investigate the impact of global and regional distribution of WMH on episodic memory and executive function in middle-aged cognitively unimpaired participants [*N* = 561 (45–75 years)] enriched for Alzheimer’s disease risk factors. WMH were automatically segmented from FLAIR, T1 and FSE MR images. WMH load was calculated both globally and regionally. At each cerebral lobe, regional WMH load was measured at four equidistant layers extending from the lateral ventricles to juxtacortical areas. Cognition was measured by The Memory Binding Test (MBT) and WAIS-IV subtests. Global composite z-scores were calculated for the two cognitive domains. Association between global and regional WMH measurements were sought against cognitive measures, both in global composite scores and in individual subtests. We adjusted cognition and WMH burden for the main sociodemographic (age, sex and education) and genetic factors (*APOE-ε4*). Memory and executive function were significantly associated with global WMH load. Regionally, lower executive performance was mainly associated with higher deep WMH load in frontal areas and, to a lower degree, in occipital, parietal and temporal regions. Lower episodic memory performance was correlated with higher WMH burden in deep frontal and occipital areas. Our novel methodological approach of regional analysis allowed us to reveal the association between cognition and WMH in strategic brain locations. Our results suggest that, even a small WMH load can impact cognition in cognitively unimpaired middle-aged subjects.

**Electronic supplementary material:**

The online version of this article (10.1007/s11682-019-00151-2) contains supplementary material, which is available to authorized users.

## Introduction

White matter hyperintensities (WMH) are commonly detected through magnetic resonance imaging (MRI) in the brain of elderly individuals (Longstreth et al. 1996) and are presumed to have a vascular etiology (Prins and Scheltens 2015). These lesions are typically seen after the fifth decade of life (Sachdev et al. 2005) and by 80 years of age, more than 90% of the general population has some degree of WMH (F. E. de Leeuw et al. 2001). Even though they are relatively frequent in asymptomatic individuals (Kloppenborg et al. 2014; Salvadó et al. 2019), global WMH load has been shown to exert a negative impact on multiple cognitive domains (Bolandzadeh et al. 2012; Lampe et al. 2017; Prins et al. 2005), on the onset and severity of dementia (Habes et al. 2016), and to constitute an independent risk factor for cognitive decline (Bolandzadeh et al. 2012). Mainly, they have been associated with frontal dysfunction including impairments in attention, executive function and processing speed (Desmond 2002; Kloppenborg et al. 2014). Furthermore, severe WMH burden has been associated to the risk of progressing from normal cognition to mild cognitive impairment (MCI) and to contribute to the severity of dementia syndromes (Eric E. Smith et al. 2008). As stated in the study from the Alzheimer’s Disease Neuroimaging Initiative (ADNI), WMH could be an important predictor of subsequent short-term global cognitive decline (Carmichael and Schwarz 2010). Brickman et al. (2012) demonstrated that increased WMH burden in parietal regions increases the risk of developing Alzheimer’s Dementia in cognitively normal older adults (Brickman et al. 2012). Additionally, previous studies have shown that occurrence of WMH are associated, in cognitively unimpaired individuals, to Alzheimer’s disease (AD) risk factors (Salvadó et al. 2019), including the presence of two *APOE*-ε4 alleles, the strongest genetic risk factor for AD (Rojas et al. 2017).

On the other hand, fewer studies have investigated the impact of the topographical location of WMHs to better depict the impact on cognition of lesions in strategic brain areas (Bolandzadeh et al. 2012; Desmond 2002; Luo et al. 2017). Existing literature supports an association between cognitive impairment and periventricular WMH over juxtacortical/deep WMH (Bolandzadeh et al. 2012). Periventricular WMH affect areas with high density of long associating tracts that connect the cortex with subcortical and other more distant cortical areas, whereas juxtacortical white matter contains more short-associating fibers (Jan Cees De Groot et al. 2002).

In addition to these, previous studies support that, in cognitively healthy participants, WMHs at strategic brain locations are also associated with cognitive decline, particularly affecting executive function (Ramirez et al. 2014), information processing speed (Jacobs et al. 2013; Luo et al. 2017) and memory (Ramirez et al. 2014). Murray et al. (2010), used a region of interest approach and found that higher WMH load in all regions, except the occipital lobe, was correlated with lower executive function in 148 non-demented elderly participants with a median age of 79 years (Murray et al. 2010). De Groot et al. (2002) reported that both visually rated periventricular and subcortical WMH were associated with lower performance on processing speed on 1077 participants recruited from the general population (aged between 60 and 90 years). A cross-sectional study with 100 asymptomatic participants (mean age 59.7 years) found that higher burden on deep WMH was associated with significantly lower scores in executive function, attention, verbal fluency, visual memory, visuospatial skills and psychomotor speed (Soriano-Raya et al. 2012). In a recent ROI-based study, Jiang et al. (2018) found independent associations of regional WM lesions on strategic WM tracts in processing speed and executive function in a community-dwelling sample of older individuals (mean age 78.26 years) (Jiang et al. 2018).

There is scarce literature reporting the association between regional WMH load and memory performance, with discrepant results. Some previous studies with cognitively healthy participants did not find any associations between WMH load and memory performance (Arvanitakis et al. 2016; Hedden et al. 2012; Jiang et al. 2018; Murray et al. 2010). In contrast, de Groot et al. (2000) observed an association between lower performance on memory and both periventricular and subcortical WMH (J C de Groot et al. 2000). Using voxel-wise analysis, Smith et al. (2011) reported an association between posterior and periventricular WMH load and memory in a mixed sample of participants over 65 years old with AD, MCI and normal cognition (E E Smith et al. 2011). In most of these previous studies, the mean age of the participants was over 60 years old. Therefore, studies are needed to better understand the specific influence of regional WMH distribution on cognitive functioning in well-characterized middle-age individuals (Soriano-Raya et al. 2012; Wardlaw et al. 2015).

In the present study, we investigated the impact of global and regional distribution of WMH on episodic memory and executive function in a cohort of middle-aged healthy subjects. To this end, we applied a novel region of interest approach to regionally quantify WMH load and applied a statistical model to account for the contribution of known sociodemographic (age, sex, and education) and genetic factors (*APOE-ε4*) and disentangle them from the specific effect of WMH on cognitive performance.

## Methods

### Participants

The ALFA (for ALzheimer and FAmilies) cohort, established by the Barcelonaβeta Brain Research Center (BBRC), is composed by 2743 cognitively normal participants aged between 45 and 75. This cohort was established as a research platform to characterize preclinical AD in asymptomatic individuals In brief, participants had a Clinical Dementia Rating (CDR) equal to 0 and were cognitively normal as determined by a neuropsychological screening test battery that included the Mini-Mental State Examination, the Memory Impairment Screen, the Time Orientation of The Barcelona Test II and verbal semantic fluency. Individuals with presence of major psychiatric disorders or diseases that could affect cognitive abilities were excluded. In this regard, the Goldberg Anxiety and Depression Scale was used to screen for mood disorders (GADS) (Goldberg et al. 1988) (for a full description of the cohort, please refer to (Molinuevo et al. 2016)). The participants’ anthropometric measurements were measured and allowed to calculated the CAIDE (Cardiovascular Risk Factors, Aging, and Incidence of Dementia) (Kivipelto et al. 2006) dementia risk score (for more detail, please refer to (Salvadó et al. 2019)). A subgroup of 608 ALFA parent cohort participants without contraindications to brain MRI was selected to participate in the present study as a function of their *APOE* genotype, preferentially including *APOE-ε4* and *APOE–ε2* allele carriers (NCT02198586). A detailed description of the inclusion criteria for the MRI sub-study can be found in (Cacciaglia et al. 2018).

### Cognitive measures

Verbal episodic memory was evaluated through the Memory Binding Test (MBT) (Buschke 2014; Gramunt et al. 2015) which assesses immediate and delayed retention (after a lapse of 25 to 35 min) of verbal information through a controlled learning process of two different words lists. Each list contains 16 items that share the same semantic category in pairs. Both free and cued recall modalities are tested. As a form of cued recall, this test incorporates the paired recall condition, where the participant is asked to recall the two items corresponding to each of the semantic categories, so, eliciting binding. Further detail on the administration procedure of the MBT can be found in (Gramunt et al. 2015). In this study, we analysed seven MBT main outcomes corresponding to two main categories: learning and immediate recall, and delayed recall. Learning and immediate recall includes: Total Paired Recall (total of words recalled from both lists after semantic cueing); Paired Recall Pairs (number of instances when both words of the same category are successfully recalled under the paired condition); Total Free Recall (the score of immediate free recall of both lists) and Semantic Proactive Interference (calculated as a proportion of words learned of the second list with respect to the first one). Regarding delayed recall, the main outcomes are: Total Delayed Free Recall (total of words recalled under the delayed free recall condition); Total Delayed Paired Recall (total of words recalled under the delayed paired recall condition) and Pairs in Delayed Free Recall (the number of instance when both items of a semantic category are successfully recalled).

Executive function was assessed by means of four WAIS-IV subtests: the Digit span (as a measure of immediate and working memory) composed of three parts: forward, backward and sequencing; Coding subtest (as a measure of processing speed and attention); Matrix reasoning and Visual puzzles (both as measures of fluid intelligence, the former of logic and executive functioning and the latter of visual reasoning); and Similarities (a measure of abstract reasoning).

### MRI acquisition

MRI scans were acquired on a 3.0 T scanner (GE Discovery MR750 W 3 T) using the same protocol for all participants, which included one T1- and three T2- weighted sequences. The 3D-T1w sequence was designed with an isotropic voxel size of 1 mm^3^ and a matrix size of 256x256x160 (TR/TE/TI = 8.0/3.7/450 ms, NSA = 1, flip angle = 8°). Three 3D-T2w sequences, with a voxel size of 1 mm × 1 mm × 3 mm, were also used: fluid attenuation inversion recovery (FLAIR: TR/TE/TI = 11,000/90/2600 ms, flip angle = 160°), fast spin echo (FSE: TR/TE = 5000/85 ms, flip angle = 110°) and, gradient echo (GRE: TR/TE = 1300/23 ms, flip angle = 15°).

All scans were visually assessed to verify their quality and to detect incidental findings by a trained neuroradiologist and have been reported elsewhere (Brugulat-Serrat et al. 2017). In this study, ten participants were excluded due to the presence of a meningioma, as well as 37 participants due to susceptibility, motion artefacts or segmentation problems, resulting in a total of 561 images available for subsequent analysis. The medial temporal lobe atrophy was assessed by Medial Temporal Atrophy scale (Scheltens et al. 1992).

### WMH segmentation and quantification

WMH were automatically segmented using a model selection Bayesian framework (Sudre et al. 2015). In short, T1w, T2-FLAIR and T2-FSE images are rigidly coregistered using the NiftyReg package (Modat et al. 2014). The data is then modeled as a multivariate Gaussian mixture model (GMM) that simultaneously accounts for healthy tissue and unexpected observations and is constrained by subject-specific statistical tissue priors derived from the Geodesic Information Flows (GIF) algorithm (Cardoso et al. 2015).

The number of required Gaussian components is optimized on a patient level to ensure a balance between model fit and complexity. Once the model has converged, a post-processing step is applied to extract probability maps of candidate lesion voxels that are then further corrected for spurious false positive detection. Volumetric measurements are derived as the sum of this probability map over a region of interest.

**Fig. 1:**
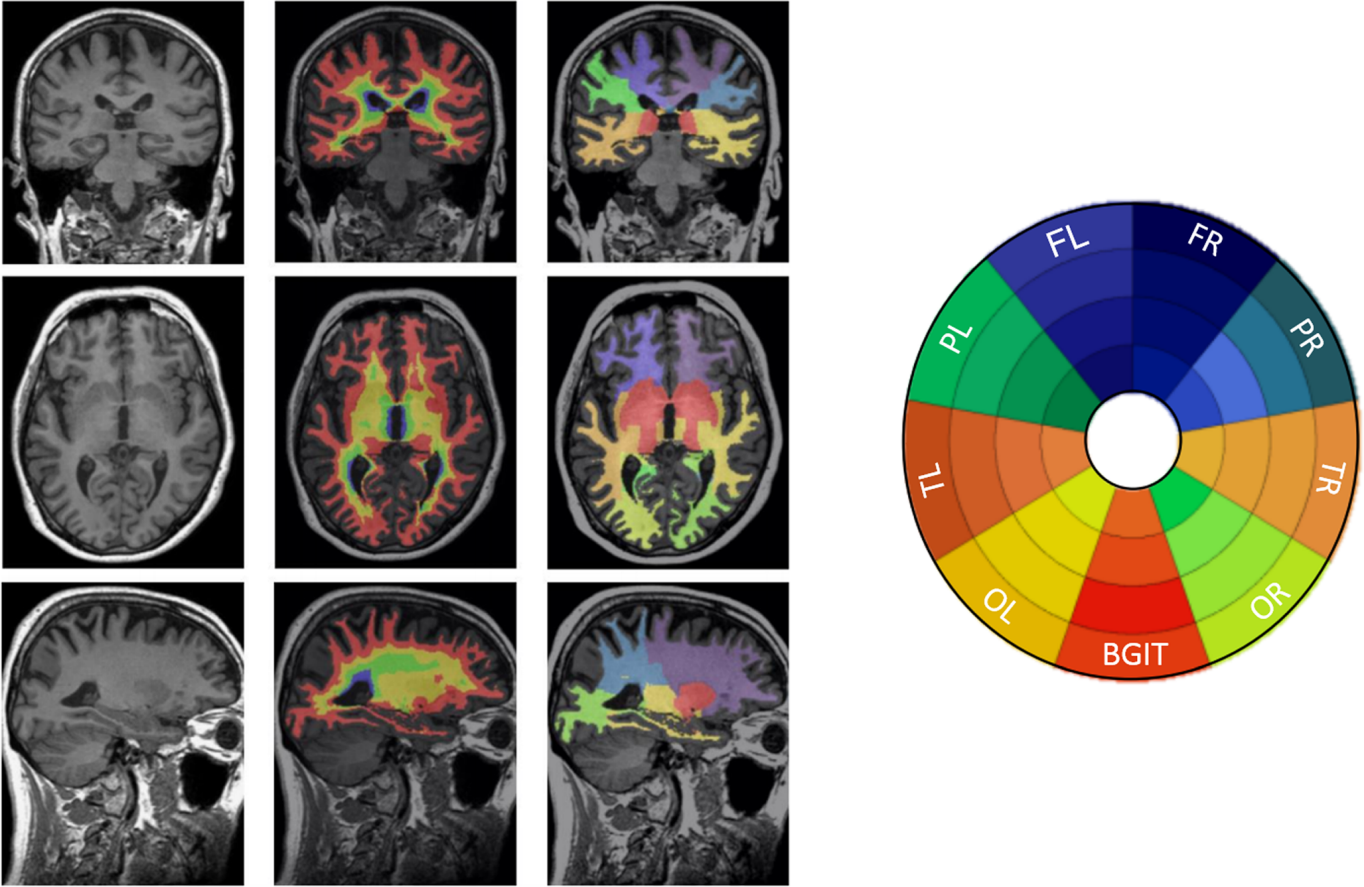
Example of the segmentation of one participant and the Bullseyes plot representation. On the left figure, the left column, T1w MR image from one participant is presented in the three views: coronal, axial and sagittal (from up to bottom). In the central and right columns, the same views are shown with the layer and lobar segmentations overlaid, respectively*.* Abbreviations: FR, frontal right; FL, frontal left; TR, temporal right; TL: temporal left; PR, parietal right; PL, parietal left; OR, occipital right; OL, occipital left and BG, basal ganglia

To depict regional results, we used a bullseye representation (C.H. Sudre et al. 2017). Every sector of the bullseyes represents one lobar white matter segment obtained based on the cortical parcellation output from the GIF algorithm. Another unique region was the basal ganglia (including internal capsule and thalamus). The concentric rings in the bullseye plot are defined by dividing the area between the ventricular surface and the cortical sheet in four equidistant layers. The interior layer in the plot represents the most periventricular area and the most external layer corresponds to the deep and juxtacortical regions. The final representation is formed of 36 regions that are composed of nine-lobar segmentation with four layers each (Fig. 1).

### Statistical analysis

First, we computed two global z-scores for the cognitive measures: memory from MBT and, executive function from WAIS-IV subtests. These global measures were calculated by averaging normalized raw scores of all subtests in each domain.

As a preliminary analysis, the cross-correlation of cognitive measures was assessed by Pearson’s correlation. Then, we sought for associations between global and regional WMH load with cognitive performance. This analysis was performed with z-scores for both global cognitive measures and for each individual subtest. Both WMH volumes and cognitive measures were adjusted by age, sex, education and *APOE* (number of *APOE-ε4* alleles). Moreover, we also adjusted WMH volumes for total intracranial volume (TIV) to correct for head size. Additionally, we replicated the analysis introducing CAIDE risk score, depression subscale of Goldberg and MTA scale as confounders.

Non-parametric tests were used (Spearman’s rank correlation) given that the distribution of WMH load heavily departed from normality (Fig. S1A) and, that none of the different transformations applied were successful to convert it to a normal distribution. A modified bootstrap method was used to calculate *p*-values. We randomly assigned the outcome variables to the original predictors 10,000 times, without resampling, and for each reassignment we calculated the Spearman’s rho statistic. Then, *p*-values were estimated by calculating the number of times the statistic was lower than the statistic calculated with the original data, and then dividing by the number of permutations done. The threshold for statistical significance was set to *p* < 0.05.

## Results

From the 608 ALFA parent cohort participants that were invited to take part in the present study, 561 provided valid MRI scans for WMH segmentation. The mean age was 57.4 years old and 60.9% were women. The main characteristics of the participants are shown in Table 1.

**Table 1:** Characteristics of the study population (*N* = 561)

Age (years), mean (SD), [range]	57.4 (7.5)
	[44–75 years]
Female sex, *n* (%)	342 (60.9)
Education (years), Mean (SD)	13.7 (3.5)
Hypertension ^a^, n (%)	147 (26.2)
Diabetes^b^, n (%)	28 (5.0%)
Hypercholesterolemia^b^, n (%)	171 (30.5)
BMI (kg/m^2^), mean (range)	26.4 (24.0–29.4)
CAIDE-I risk (%)	1.47
Number of *APOE-ε4* alleles, n (%)	
None	275 (49.0)
One *APOE-ε4* alleles	215 (38.3)
Two *APOE-ε4* alleles	71 (12.6)
TIV (10^6^ cc), median [Q1-Q3]	1420 [1330–1490]
WMH volume (10^3^mm^3^), median [Q1-Q3]	1.94 [1.13–3.69]
Medial temporal Atrophy scale (MTA), mean (SD)	0.79 (0.64)
Cognitive evaluation, Mean (SD)	
Memory binding test	
Total Paired Recall (0–32)	24.2 (4.5)
Total Free Recall (0–32)	16.6 (5.2)
Paired Recall Pairs (0–16)	9.2 (3.4)
Total Delayed Free Recall (0–32)	16.9 (5.2)
Total Delayed Paired Recall (0–32)	23.9 (4.6)
Pairs in Delayed Free Recall (0–16)	6.4 (3.1)
Semantic Proactive Interference (%)	75.2 (18.6)
WAIS-IV subtests	
Visual Puzzles (0–26)	13.3 (4.2)
Digit Span Forward (0–16)	8.5 (2.1)
Digit Span Backward (0–16)	8.0 (2.1)
Digit Span Sequencing (0–16)	8.4 (2.1)
Matrix Reasoning (0–26)	16.3 (4.3)
Similarities (0–36)	22.6 (4.7)
Coding (0–135)	65.5 (15.0)

^a^Systolic blood pressure > 140 mmHg

^b^Medications and/or self-reported

Mainly periventricular and deep frontal and parietal areas showed a greater correlations between regional and total WMH load, even all regions showed a moderate to high correlation to global WMH burden (Fig. S1B). Periventricular areas and occipital lobe are the most affected regions by WMH lesions in our cohort (Fig. S1C). However, the relative volume occupied by WMH lesions is low, as the median for the most affected regions reached only 10% of the total volume of the region.

**Fig. 2:**
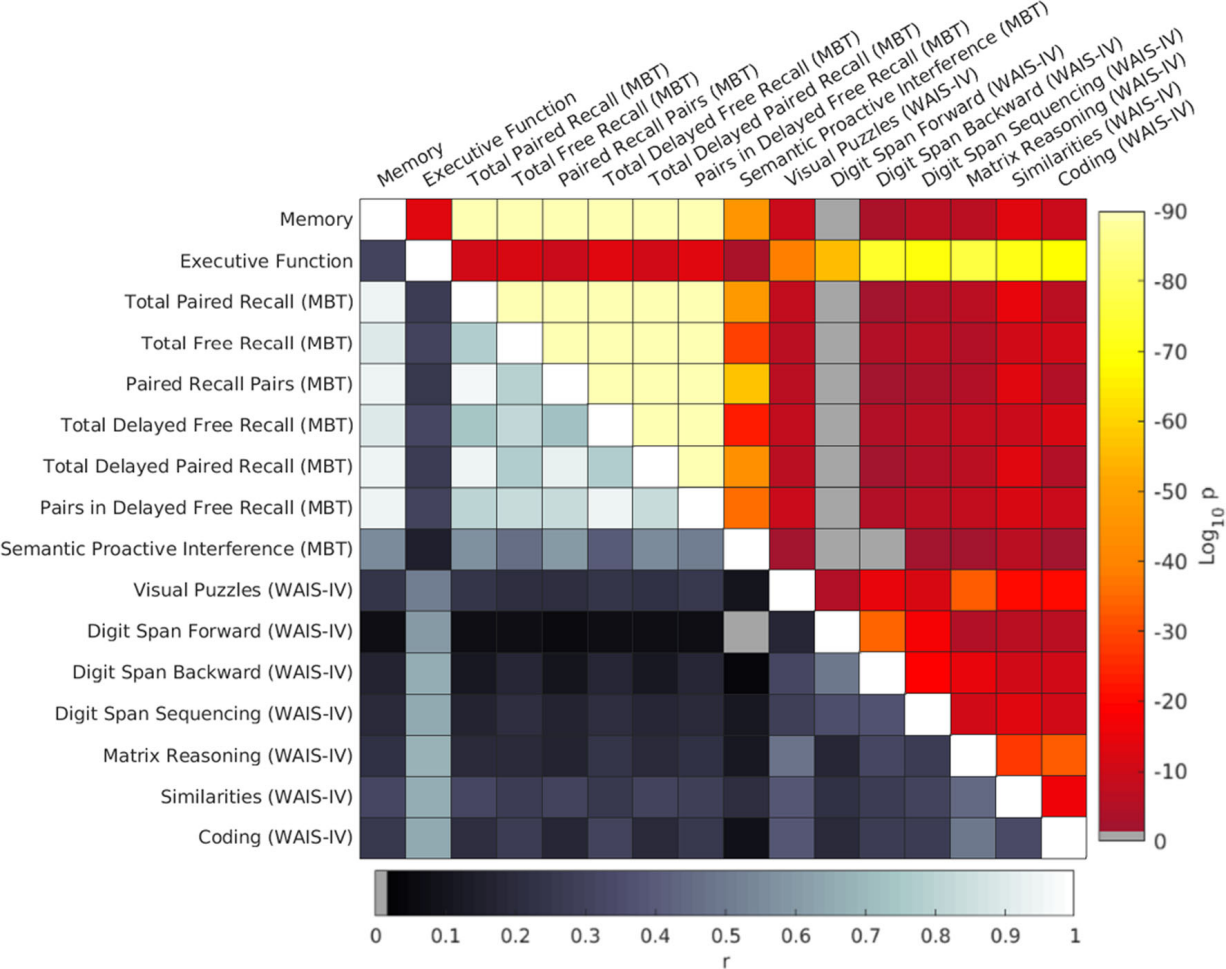
Cross-correlation matrix between pairs of the cognitive measures depicting results of Pearson’s correlations. The hot colour scale in the upper diagonal represents statistical significance (*p-values*), in which grey coloured regions depict non-significant correlations. Values down the main diagonal represent strength of correlation (*r),* which was always positive. The two cognitive domains were highly significant correlated with their respective cognitive measures. Memory measures were highly correlated among them, whereas executive function measures only showed a modest correlation among themselves but also with memory scores. Abbreviation: MBT, Memory Binding Test

Figure 2 shows the cross-correlation and statistical significance between pairs of cognitive scores. MBT subtest scores were highly correlated among them whereas the correlation between pairs of executive function scales was only modest. Logically, memory and executive function z-scores were highly correlated with their respective scales. Correlation between cognitive functions was modest but yet statistically significant (*p* < 0.001).

The effect sizes and the significance of the correlation between global WMH load and cognition after correcting by sociodemographic and genetic factors, and also TIV in global WMH burden are shown in Table 2. These values indicated that global WMH was significantly associated with lower performance on memory (Rho = −0.07, *p* = 0.045) and executive function (Rho = −0.07, *p* = 0.04). It should be noted that the effect sizes of the association with both the cognitive domains and WMH load were not strong (significant Rho values were below 0.2).

**Table 2:** Correlation between global WMH load and cognition

	Rho [95% CI]	*p*
Cognitive z-score composites		
Memory	−0.07 [−0.156–0.001]	0.04*
Executive function	−0.07 [−0.152–0.012]	0.04*
Memory binding test		
Total free recall	−0.05 [−0.131–0.029]	0.12
Total delayed free recall	−0.07 [−0.156–0.001]	0.04*
Total paired recall	−0.05 [−0.130–0.037]	0.14
Total delayed paired recall	−0.07 [−0.155 - -0.001]	0.04*
Paired recall pairs	−0.06 [−0.146–0.019]	0.07^¥^
Pairs in delayed free recall	−0.08 [−0.166 - -0.001]	0.03*
Semantic proactive interference	−0.08 [−0.161–5.5e-05]	0.03*
Subtests of WAIS-IV		
Digit span forward	−0.01[−0.090–0.079]	0.42
Digit span backward	−0.09 [−0.174 - -0.001]	0.01*
Digit span sequencing	0.03 [−0.056–0.110]	0.28
Coding	−0.06 [−0.135–0.023]	0.08^¥^
Visual puzzles	−0.01 [−0.095–0.070]	0.39
Matrix reasoning	−0.05 [−0.129–0.031]	0.12
Similarities	−0.06 [−0.143–0.024]	0.08^¥^

Adjusted by age, sex, education and number of *APOE-****ε****4* alleles. WMH also adjusted by TIV.

*p < 0.05

^¥^Significant trend (*p* ≤ 0.1)

**Fig. 3:**
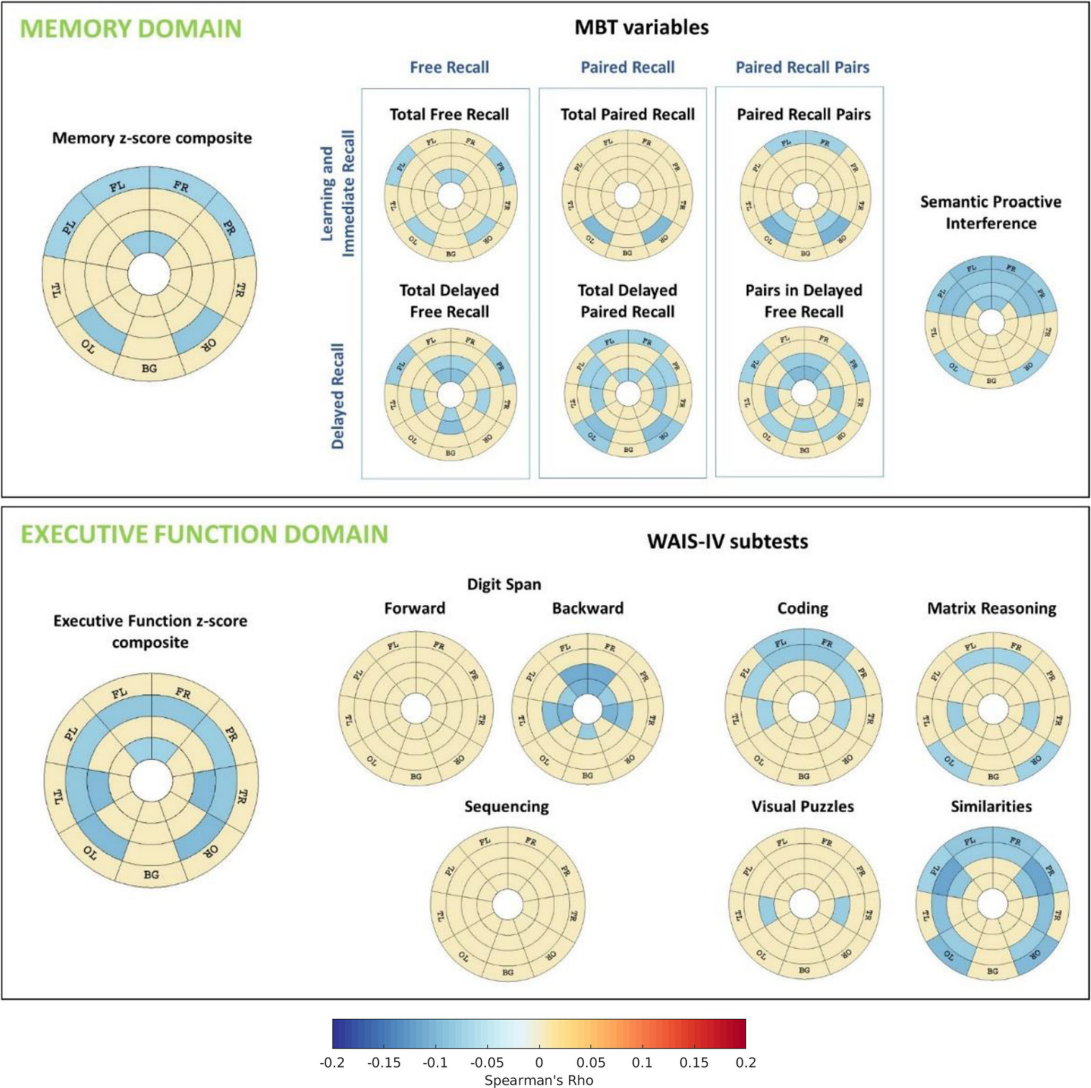
Regional patterns of correlation between cognition and WMH. Effect sizes of these correlation measures by Spearman’s Rho are shown in those areas that presented statistical significant association (*p* < 0.05). All the other regions are depicted in beige. Cognition and WMH were adjsuted by age, sex, education and number of *APOE-ε4* alleles. WMH were also adjusted by TIV. Abbreviations: FR, frontal right; FL, frontal left; TR, temporal right; TL: temporal left; PR, parietal right; PL, parietal left; OR, occipital right; OL, occipital left; BG, basal ganglia; WMH, white matter hyperintensities; TIV, total intracranial volume

Figure 3 shows the regional patterns of correlation between cognition and WMH, adjusted by age, sex, education and number of *APOE-ε4* alleles, as well as by TIV in the case of WMH. As expected, all significant correlations were negative (i.e. the higher the WMH burden, the lower the cognitive performance). Lower performance on the executive function z-score composite was related to higher WMH load mainly in deep WM, except for the periventricular frontal region. With respect to the memory z-score composite, lower performance was significantly associated with WMH load in the periventricular frontal region and in deep frontal, parietal and occipital regions.

Regarding executive function subtests (Fig. 3), lower performance was correlated mainly with deep frontal WMH and with parietal and occipital areas. Lower learning and immediate recall were associated with a pattern of higher WMH load in frontal, parietal and occipital areas. Specifically, deep WMH in occipital regions were related to lower paired recall. Finally, lower delayed recall was related to WMH load in periventricular frontal regions and in deep temporal, parietal and occipital areas.

Finally, when we introduced as a confounders: MTA scale (Table S1, Fig. S2), CAIDE-I (Table S2, Fig. S3) or depression subscale of Goldberg (Table S3, Fig. S4), we did not find any significant change with the results previously exposed (neither in the global nor regional analysis).

## Discussion

In the present study, we explored the relationship of global and regional WMH load distribution versus cognitive performance in middle-aged cognitively unimpaired participants, showing that even a small WMH load can impact cognition. This study adds to the small number of studies showing associations between cognitive function and the spatial distribution of WMH in healthy subjects (Bolandzadeh et al. 2012; Desmond 2002). Here, we report regional patterns of WMH burden related to lower performance on memory an executive function after correcting by age, sex, education and *APOE-ε4*.

Both global and regional WMH load were significantly associated with lower executive functioning and memory z-score composites. Regionally, deep WMH burden in all lobes was associated with lower executive functioning, whereas lower memory performance was significantly associated with higher WMH load in juxtacortical frontal, parietal, and occipital areas, as well as in periventricular frontal regions.

In agreement with previous studies (Bolandzadeh et al. 2012), we found that periventricular WMH load was associated with lower cognitive performance. These studies suggest that periventricular WMH may have a stronger negative impact on cognitive performance than juxtacortical/deep WMH, probably due to the disruption of long association tracts (J C de Groot et al. 2000; Debette et al. 2007; Kim et al. 2011; Söderlund et al. 2006). In our work, however, we found that deep WMH regions were more strongly associated with both cognitive domains and most of the cognitive variables analysed. These findings suggest that the association between periventricular WMH load and cognition found in previous studies might be mediated by age or other risk factors for WMH. Therefore, their impact on cognition might not entirely be mediated by WMH burden, as might be with deep WMH load (F.-E. de Leeuw et al. 2002; Ylikoski et al. 1995).

In our study, deep WMH load in all lobes was associated with lower scores in the executive function composite. These results are in line with previous research that found a significant association of WMH in frontal (J C de Groot et al. 2000; Murray et al. 2010; E E Smith et al. 2011; Soriano-Raya et al. 2012; Tullberg et al. 2004), temporal (Jiang et al. 2018; E E Smith et al. 2011), parietal (Murray et al. 2010; E E Smith et al. 2011; Tullberg et al. 2004) and occipital regions (Jiang et al. 2018; E E Smith et al. 2011; Tullberg et al. 2004) with lower performance in executive function. Notwithstanding, only one study reported the association between deep WMH and executive functions (Soriano-Raya et al. 2012). Hence, our findings support for a significant negative impact of deep WMH on executive function performance, even in cognitively healthy middle-aged individuals.

Processing speed has been consistently related to WMH (Bolandzadeh et al. 2012; J C de Groot et al. 2000; Jiang et al. 2018; Pantoni et al. 2007; Strauss et al. 2006). We found a correlation between a pattern of higher WMH load in deep frontal, parietal and temporal regions and visuomotor processing speed (measured by Coding). This result is in line with previous studies that shown that processing speed deficits can be explained by parietal WMH volume, age and their interaction (Jacobs et al. 2013; Luo et al. 2017).

With respect working memory, the specific regional WMH pattern was consistent with previous studies showing that periventricular and deep WMH volume, in temporal and frontal regions, were correlated with poorer performance on working memory assessed by the Digit Span Backward (Charlton et al. 2010; Oosterman et al. 2008). By contrast, other studies could not detect this association (Murray et al. 2010; Oosterman et al. 2004, 2008; Sachdev et al. 2005; Schmidt et al. 1993; Skoog et al. 2009). These inconsistent results may be explained in part by differences in the mean age of the included participants in these studies in addition to the strong association between WMH with other confounders. We did not find any significant correlation between the Digit Span Forward and Sequencing and regional WMH load. These differences between Digit Span Forward and, Sequencing and Backward results may be related to the main underlying cognitive process. While Digit Span Forward and Sequencing mainly rely on short-term ability, Backward Condition is a more complex task that involves higher working memory component (Charlton et al. 2010; Gerton et al. 2004; Lezak et al. 2012).

Regarding abstract reasoning performance, WMH load had a specific contribution in a broad regional pattern involving deep and juxtacortical regions. Au et al. (2006) did not find differences in abstract reasoning performance (assessed also by means of the Similarities subtest) when dichotomizing between higher and lower WMH load. In comparison to our study, the age range of the studied population was much wider (34–88) and the impact on WMH load of sociodemographic and genetics factors was not accounted for (Au et al. 2006). We also found that lower performance in Visual Puzzles and Matrix Reasoning were associated with higher deep and juxtacortical WMH in frontal, temporal, and occipital regions and in deep temporal regions respectively. This result can be attributed to the involvement of non-verbal fluid reasoning from both tests (Lezak et al. 2012; Ward et al. 2012), which is strongly influenced by age (Der et al. 2010). Given the scarce literature on this WAIS-IV subtest, our results contribute to the understanding of the impact of specific regional WMH patterns on it.

We found that lower performance in the memory composite was associated with a higher load of juxtacortical WMH in frontal and parietal regions, in deep occipital regions and, lastly, in periventricular frontal areas. This finding supports previous research with older non-demented participants in which memory performance, as assessed by word verbal learning and/or story recall, was associated with periventricular (Burns et al. 2005; Tullberg et al. 2004) and subcortical WMH load (J C de Groot et al. 2000). Finally, in line with previous studies (J C de Groot et al. 2000; E E Smith et al. 2011), it could be suggested that deep WMH in occipital regions are a specific regional WMH pattern associated with a lower performance on memory.

Regarding the MBT outcomes, a higher volume of global WMH was associated to lower performance in the outcomes that correspond to the delayed recall category. This result supports evidence from previous work which supported WMH to be the strongest structural predictor of episodic memory delayed-recall performance (Petkov et al. 2004).

Our results suggest that deep WMH in occipital lobes have a significant negative impact on memory binding. This result is in line with a previous study that examined the neural correlates of semantic associative encoding by a similar procedure as the MBT (Lepage et al. 2000). In this report, it was found that multiple posterior brain regions, such as the inferior portion occipital-temporal cortices, were activated with this cognitive process. It could be speculated that WMH in these areas might influence semantic association process, previously cited as binding, resulting in a reduced capability to benefit from the shared semantic category clue both in immediate and delayed recall.

As for the specific negative impact of WMH on the performance of the other MBT variables, the results of this study show a specific spatial pattern of higher WMH associated with lower scores in learning and immediate recall in mainly periventricular frontal and occipital regions. In contrast to previous studies (Ramirez et al. 2014; E E Smith et al. 2011), we did not find a correlation in temporal regions. Nevertheless, in these, the correlation was found in mixed samples (cognitively normal, MCI and AD) (E E Smith et al. 2011) or only AD participants (Ramirez et al. 2014). This suggests that a higher level of WMH affectation is needed in these areas may to impact learning and immediate recall. Regarding delayed recall, we found an association with WMH load and lower scores in anterior periventricular frontal areas, adjacent to the caudate, and in basal ganglia. These results further support the participation of basal ganglia in associative learning (Scimeca and Badre 2012; Sheth et al. 2011). It has been suggested that caudate plays an intermediary role in information transfer between the hippocampus and prefrontal nodes implicated in retrieval control processes (Geib et al. 2017). Also in line with existing literature (Daselaar and Cabeza 2008; Feredoes et al. 2006), we found that high semantic proactive interference was mainly associated to higher juxtacortical and deep WMH load in frontal and parietal.

The main contribution of our work is the study of the impact of regional WMH burden on cognitive performance in middle-aged healthy participants. This is a relatively young and healthy sample in comparison with most of the existing literature. Another strength is that the administration of novel cognitive tests in this study contributes to the scarce literature on relevant factors driving their performance. Finally, the bullseye’s regional representation of WMH allows differentiating periventricular, deep and juxtacortical lesions per lobe (C.H. Sudre et al. 2017).

Nevertheless, there are also some limitations in our study. The selection of participants preferably by having at least one *APOE-ε4* or *APOE-ε2* allele can constitute a selection bias. However, the analyses were repeated removing the confounding effect of *APOE* obtaining similar results. We can, therefore, conclude that the recruitment criteria have not driven the bulk of our results findings and that our results can be extrapolated to the general population. Another limitation resides in the use of non-parametric tests may have lowered the level of statistical power to detect significant associations. The high percentage of relatively young and WMH-free individuals resulted in a right-skewed distribution of WMH load (Fig. S1A of Supplementary Material), which prevented the use of parametric statistics. Another obvious limitation is the cross-sectional nature of present study. The longitudinal follow-up will allow assessing the regional patterns of WMH load that are associated to cognitive decline. Further, it has been suggested that WMH load might interact with the hallmarks of AD pathology, such as abnormal depositions of β-amyloid (Aβ) and tau-protein. We expect that the impact of tau on our results to be minimal, given that we studied a cognitively healthy sample and tau abnormalities are tightly associated with cognitive decline (Jansen et al. 2018). On the other hand, Aβ abnormalities have been widely described in cognitively healthy individuals (Jansen et al. 2018). Given that our sample is enriched for *APOE-ε4* carriers and since Aβ biomarkers were not available in this study, we cannot rule out an impact in our results of a potentially higher prevalence of Aβ positivity. Future studies will include core AD biomarkers to assess their contribution to WMH load and their joint impact on cognition. Previous reports suggest that amyloid and WMH load are independent pathologic processes that exert independent effects on brain health (Roseborough et al. 2017). However, a recent study shows that WMH volume is highly associated with preclinical AD (Kandel et al. 2016). Since characterization of Aβ the cohort here studied is ongoing, we expect to be able to contribute to this discussion in short.

In conclusion, we found a significant impact of deep WMH load on cognitive performance in middle-aged cognitively-healthy individuals after accounting for known confounders. Lower executive function was related to higher WMH load mainly in deep WM areas after adjusting for known confounders. Association with lower episodic memory performance was found in frontal and occipital areas, specifically related to lower paired recall. Therefore, our novel methodological approach of regional WM analysis load has proven to be useful to reveal the association between cognition and WMH in strategic brain regions. Our results extend on previous reports in older populations to younger individuals who could potentially benefit from primary prevention strategies.

## Electronic supplementary material

ESM 1 (PDF 706 kb)
