# Supplementary material for: Patterns of white matter hyperintensities associated with cognition in middle-aged cognitively healthy individuals
Source: Brain Imaging Behav. 2019 Jul 5;14(5):2012–23. doi: 10.1007/s11682-019-00151-2 (PMC7572336; doi:10.1007/s11682-019-00151-2)
Supplement: Supplementary file 1 — (PDF 706 kb) [file 11682_2019_151_MOESM1_ESM.pdf]

Supplementary Material

PATTERNS OF WHITE MATTER HYPERINTENSITIES ASSOCIATED WITH COGNITION IN MIDDLE-AGED COGNITIVELY HEALTHY INDIVIDUALS

Anna Brugulat-Serrat[1*], Gemma Salvadó[1*], Carole H. Sudre[2,3], Oriol Grau-Rivera[1], Marc Suárez-Calvet[1], Carles Falcon[1,4], Gonzalo Sánchez-Benavides[1], Nina Gramunt[1], Karine Fauria[1], M. Jorge Cardoso[2,3], Frederik Barkhof[5,6,7], José Luis Molinuevo[1,8§], Juan Domingo Gispert[1,4,9 §], for the ALFA Study[†]

1) Barcelonaβeta Brain Research Center, Pasqual Maragall Foundation, Barcelona, Spain
2) School of Biomedical Engineering and Imaging Sciences, King's College London, London, UK,
3) Dementia Research Centre, UCL, London, UK,
4) Centro de Investigación Biomédica en Red de Bioingeniería, Biomateriales y Nanomedicina (CIBER-BBN), Madrid, Spain
5) Translational Imaging Group, CMIC, UCL, London, UK
6) Brain Repair and Rehabilitation, UCL Institute of Neurology, London, UK,
7) Radiology & Nuclear Medicine, VU University Medical Centre, Amsterdam, Netherland,
8) Centro de Investigación Biomédica en Red de Fragilidad y Envejecimiento Saludable (CIBERFES), Madrid, Spain
9) Universitat Pompeu Fabra, Barcelona, Spain

[*] These authors contributed equally to this article
[†]The complete list of collaborators of the ALFA Study can be found in the acknowledgements section

§ Corresponding authors:

Juan Domingo Gispert López

Barcelonaβeta Brain Research Center and Pasqual Maragall Foundation, Wellington 30, 08003 Barcelona, Spain. Tel.: +34 933160990; Fax: +34 932275783. E-mail: jdgispert@barcelonabeta.org

José Luis Molinuevo

Barcelonaβeta Brain Research Center and Pasqual Maragall Foundation, Wellington 30, 08003 Barcelona, Spain. Tel.: +34 933160990; Fax: +34 932275783. E-mail: jlmolinuevo@barcelonabeta.org

Table S1. Correlation between global WMH load and cognition		
	**Rho [95% CI]**	***p***
**Cognitive z-score composites**		
Memory	-0.07 [-0.151 - 0.012]	0.04
Executive Function	-0.07 [-0.151 - 0.012]	0.04
**Memory Binding Test**		
Total Free Recall	-0.05 [-0.132 - 0.033]	0.12
Total Delayed Free Recall	-0.07 [-0.151 - 0.012]	0.05
Total Paired Recall	-0.05 [-0.132 - 0.033]	0.13
Total Delayed Paired Recall	-0.07 [-0.151 - 0.012]	0.04
Paired Recall Pairs	-0.06 [-0.142 - 0.022]	0.07
Pairs in Delayed Free Recall	-0.08 [-0.161 – 0.002]	0.02
Semantic Proactive Interference	-0.08 [-0.161 – 0.002]	0.02
**Subtests of WAIS-IV**		
Digit Span Forward	-0.01[-0.092 – 0.072]	0.43
Digit Span Backward	-0.09 [-0.171 - -0.008]	0.01
Digit Span Sequencing	0.03 [-0.052 - 0.112]	0.28
Coding	-0.06 [-0.142 - 0.022]	0.09
Visual Puzzles	-0.01 [-0.092 - 0.072]	0.39
Matrix Reasoning	-0.05 [-0.132 - 0.033]	0.12
Similarities	-0.06 [-0.142 - 0.022]	0.07

Adjusted by age, education, sex, number of APOE-ε4 alleles and MTA. WMH also adjusted by TIV.

Table S1. Correlation between global WMH load and cognition correcting by age, education, sex, number of APOE-ε4 alleles, TIV and MTA. Abbreviations: TIV, total intracranial volume; MTA, medial temporal atrophy.

**Table S2. Correlation between global WMH load and cognition**		
	**Rho [95% CI]**	***p***
**Cognitive z-score composites**		
Memory	-0.07 [-0.151 – 0.012]	0.04
Executive Function	-0.07 [-0.151 - 0.012]	0.05
**Memory Binding Test**		
Total Free Recall	-0.05 [-0.132 - 0.033]	0.11
Total Delayed Free Recall	-0.07 [-0.151 - 0.012]	0.04
Total Paired Recall	-0.05 [-0.132 - 0.033]	0.13
Total Delayed Paired Recall	-0.07 [-0.151 – 0.102]	0.03
Paired Recall Pairs	-0.06 [-0.142 - 0.022]	0.06
Pairs in Delayed Free Recall	-0.09 [-0.171 - -0.008]	0.02
Semantic Proactive Interference	-0.08 [-0.161 – 0.002]	0.03
**Subtests of WAIS-IV**		
Digit Span Forward	-0.01[-0.092 – 0.072]	0.40
Digit Span Backward	-0.09 [-0.171 - -0.008]	0.02
Digit Span Sequencing	0.04 [-0.042 - 0.122]	0.21
Coding	-0.06 [-0.142 - 0.022]	0.08
Visual Puzzles	-0.01 [-0.092 - 0.072]	0.38
Matrix Reasoning	-0.05 [-0.132 - 0.033]	0.13
Similarities	-0.05 [-0.132 - 0.033]	0.13

Adjusted by age, education, sex, number of APOE-ε4 alleles and CAIDE risk score. WMH also adjusted by TIV.

Table S2. Correlation between global WMH load and cognition correcting by age, education, sex, number of APOE-ε4 alleles, TIV and CAIDE. Abbreviations: TIV, total intracranial volume; CAIDE, Cardiovascular Risk Factors, Aging, and Incidence of Dementia.

Table S3. Correlation between global WMH load and cognition

	Rho [95% CI]	*p*
**Cognitive z-score composites**		
Memory	-0.08 [-0.161 – 0.002]	0.03
Executive Function	-0.07 [-0.151 - 0.012]	0.05
**Memory Binding Test**		
Total Free Recall	-0.05 [-0.132 - 0.033]	0.11
Total Delayed Free Recall	-0.07 [-0.151 - 0.012]	0.05
Total Paired Recall	-0.06 [-0.142 - 0.022]	0.09
Total Delayed Paired Recall	-0.08 [-0.161 – 0.002]	0.03
Paired Recall Pairs	-0.07 [-0.151 - 0.012]	0.05
Pairs in Delayed Free Recall	-0.09 [-0.171 - -0.008]	0.01
Semantic Proactive Interference	-0.08 [-0.161 – 0.002]	0.03
**Subtests of WAIS-IV**		
Digit Span Forward	-0.01[-0.092 – 0.072]	0.40
Digit Span Backward	-0.09 [-0.171 - -0.008]	0.02
Digit Span Sequencing	0.02 [-0.062 - 0.102]	0.31
Coding	-0.06 [-0.142 - 0.022]	0.08
Visual Puzzles	-0.01 [-0.092 - 0.072]	0.38
Matrix Reasoning	-0.05 [-0.132 - 0.033]	0.13
Similarities	-0.05 [-0.132 - 0.033]	0.11

Adjusted by age, education, sex, number of APOE-ε4 alleles and Goldberg depression subscale. WMH also adjusted by TIV.

Table S3. Correlation between global WMH load and cognition correcting by age, education, sex, number of APOE-ε4 alleles, TIV and Goldberg depression subscale. Abbreviations: TIV, total intracranial volume.

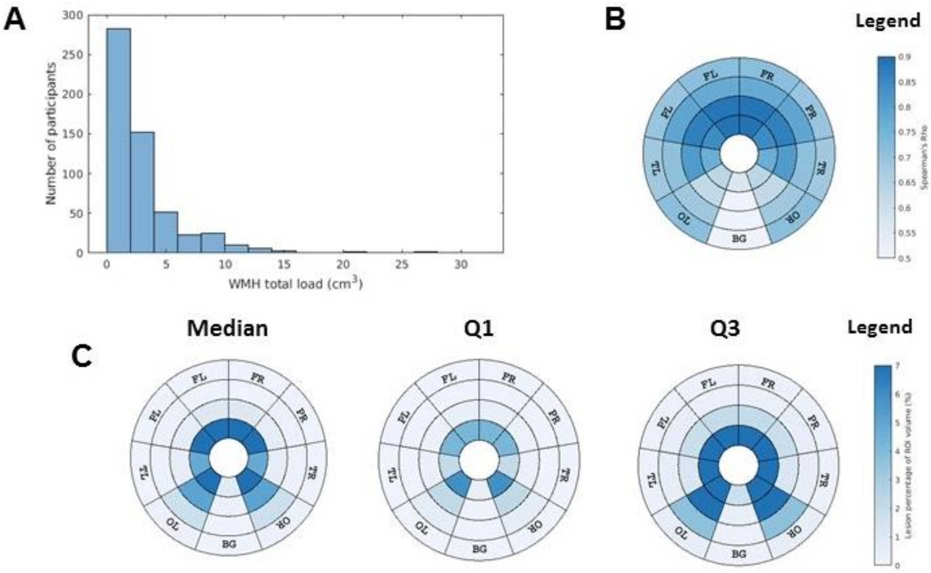

Figure S1. A) Frequency Distribution of WMH load for all the participants in the analyses (N=561) B) Correlation between global and regional WMH lesion C) WMH percentage of ROI volume in each region.

FR: frontal right, FL: frontal left, TR: temporal right, TL: temporal left, PR: parietal right, PL: parietal left, OR: occipital right, OL: occipital left, BG: basal ganglia, Q1: first interquartile and Q3: third interquartile.

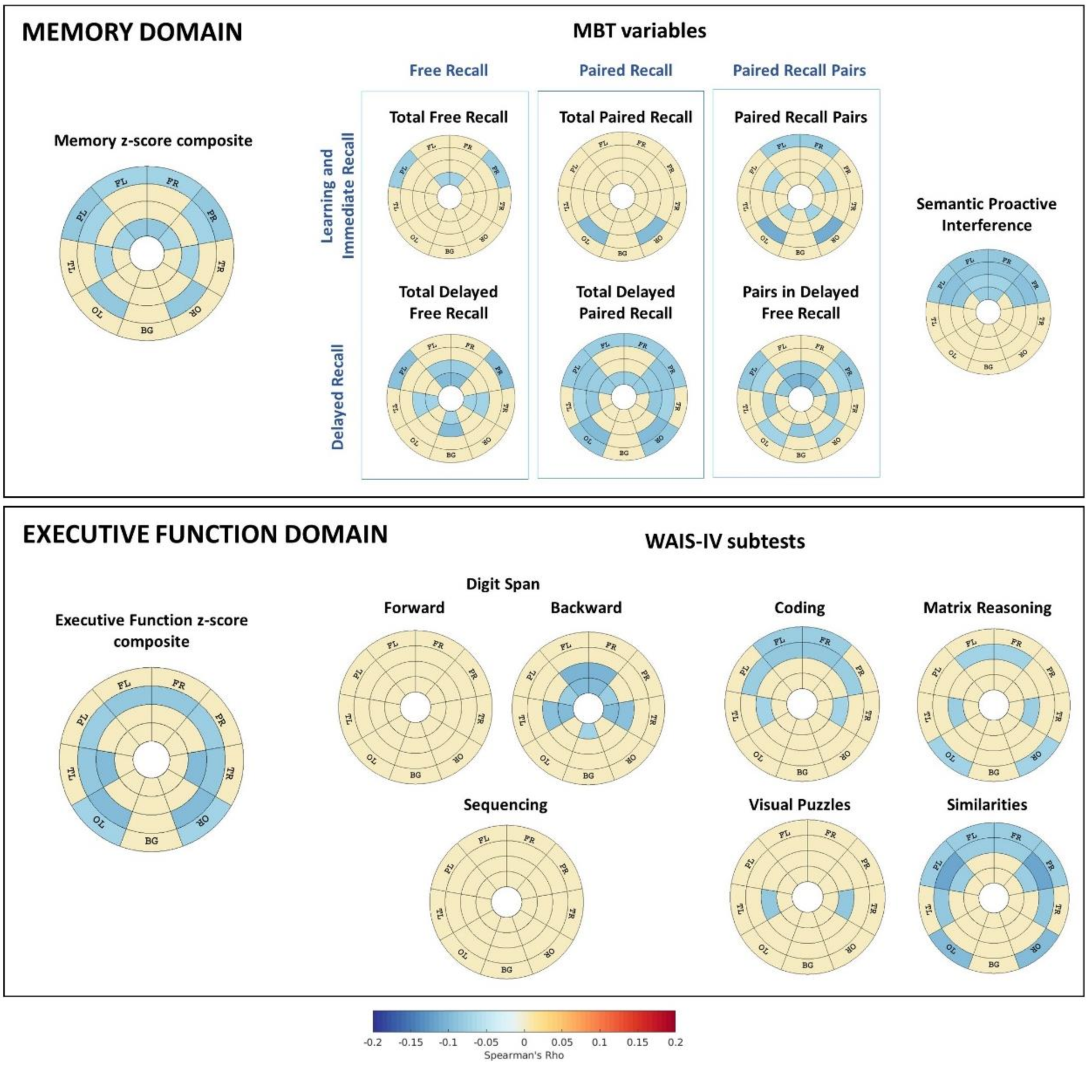

Fig. S2: Regional patterns of correlation between cognition and WMH correcting by age, education, sex, number of APOE-ε4 alleles, TIV and MTA. Effect sizes of these correlation measures by Spearman's Rho are shown in those areas that presented statistical significant association ($p<0.05$). All the other regions are depicted in beige. Cognition and WMH were adjsuted by age, sex, education, number of *APOE-ε4* alleles and MTA. WMH were also adjusted by TIV.

Abbreviations: FR, frontal right; FL, frontal left; TR, temporal right; TL: temporal left; PR, parietal right; PL, parietal left; OR, occipital right; OL, occipital left; BG, basal ganglia; WMH, white matter hyperintensities; MTA, medial temporal atrophy; TIV, total intracranial volume.

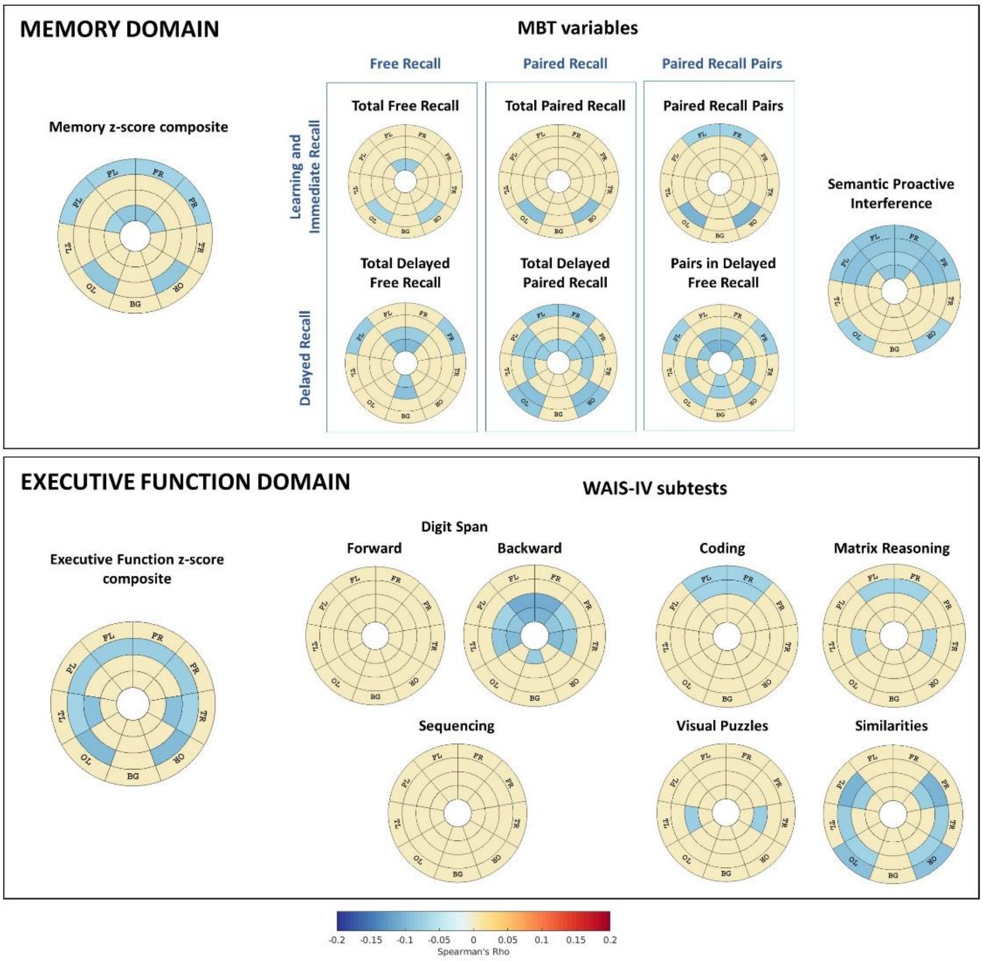

Fig. S3: Regional patterns of correlation between cognition and WMH correcting by age, education, sex, number of APOE-ε4 alleles, TIV and CAIDE-I. Effect sizes of these correlation measures by Spearman's Rho are shown in those areas that presented statistical significant association ($p<0.05$). All the other regions are depicted in beige. Cognition and WMH were adjsuted by age, sex, education, number of *APOE-ε4* alleles and CAIDE-I. WMH were also adjusted by TIV.

Abbreviations: FR, frontal right; FL, frontal left; TR, temporal right; TL: temporal left; PR, parietal right; PL, parietal left; OR, occipital right; OL, occipital left; BG, basal ganglia; WMH, white matter hyperintensities; CAIDE, Cardiovascular Risk Factors; TIV, total intracranial volume.

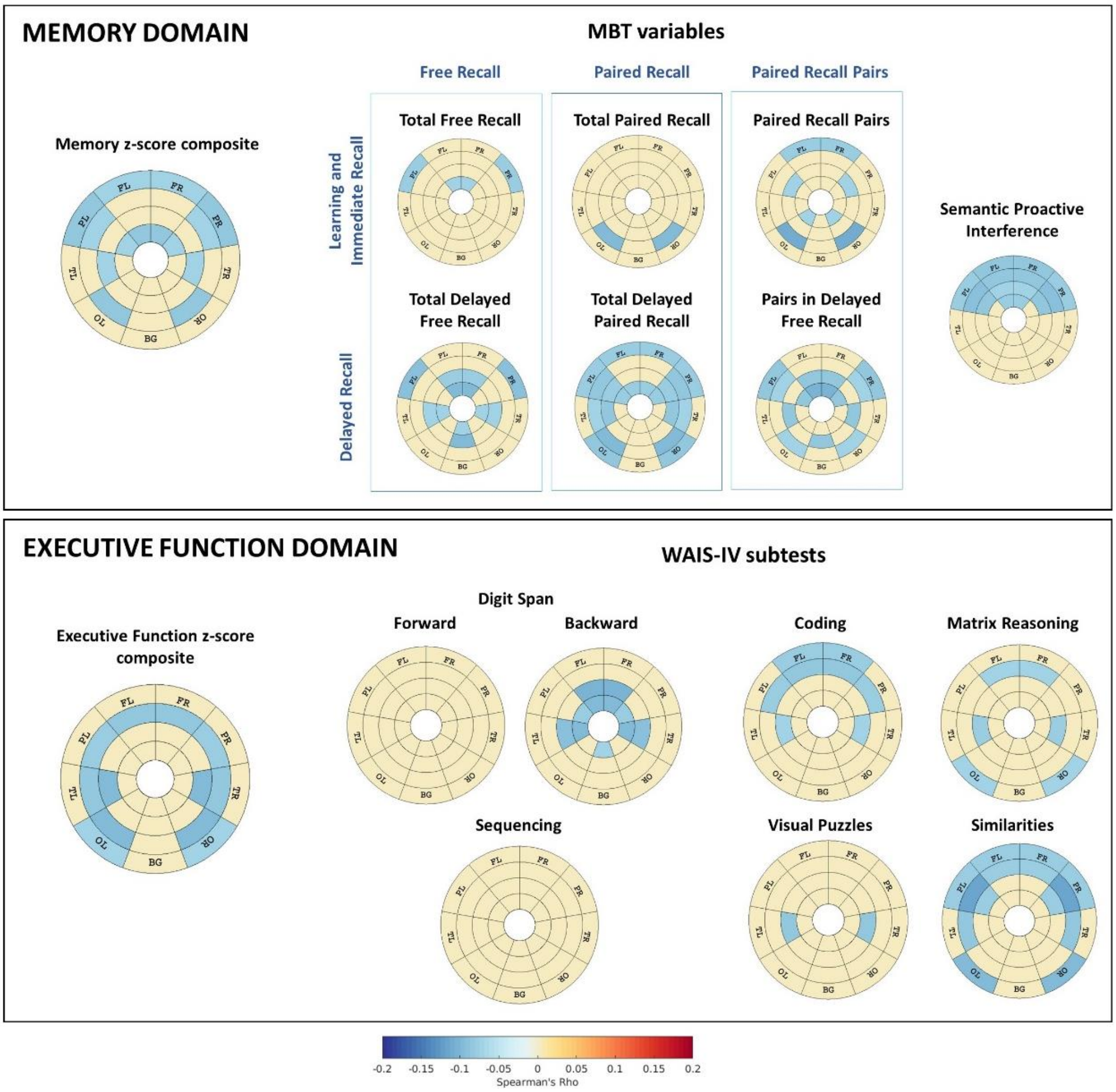

Fig. S4: Regional patterns of correlation between cognition and WMH correcting by age, education, sex, number of APOE-ε4 alleles, TIV and Goldberg depression subscale. Effect sizes of these correlation measures by Spearman's Rho are shown in those areas that presented statistical significant association (p<0.05). All the other regions are depicted in beige. Cognition and WMH were adjsuted by age, sex, education, number of *APOE-ε4* alleles and Goldberg depression subscale. WMH were also adjusted by TIV.

Abbreviations: FR, frontal right; FL, frontal left; TR, temporal right; TL: temporal left; PR, parietal right; PL, parietal left; OR, occipital right; OL, occipital left; BG, basal ganglia; WMH, white matter hyperintensities; CAIDE, Cardiovascular Risk Factors; TIV, total intracranial volume.
